# Protective Action of *Carica papaya* on β-Cells in Streptozotocin-Induced Diabetic Rats

**DOI:** 10.3390/ijerph13050446

**Published:** 2016-04-27

**Authors:** Pedro H. Miranda-Osorio, Andrés E. Castell-Rodríguez, Juan Vargas-Mancilla, Carlos A. Tovilla-Zárate, Jorge L. Ble-Castillo, Dora E. Aguilar-Domínguez, Isela E. Juárez-Rojop, Juan C. Díaz-Zagoya

**Affiliations:** 1 Centro de Investigación, División Académica de Ciencias de la Salud, Universidad Juárez Autónoma de Tabasco (UJAT), Villahermosa, Tabasco 86100, Mexico; ph.miranda@live.com.mx (P.H.M.-O.); jorge.ble@ujat.mx (J.L.B.-C.); dorisis852@hotmail.com (D.E.A.-D.); 2 División de Investigación, Facultad de Medicina, Universidad Nacional Autónoma de Mexico, Mexico, D.F., 04510, México; castell@unam.mx (A.E.C.-R.); zagoya@unam.mx (J.C.D.-Z.); 3 Unidad Profesional Interdisciplinaria de Ingeniería, Instituto Politécnico Nacional, Silao de Victoria, Guanajuato 36275, Mexico; dr.ju.vargas@gmail.com; 4 División Académica Multidisciplinaria de Comalcalco, Universidad Juárez Autónoma de Tabasco, Comalcalco, Tabasco 86650, Mexico; alfonso_tovilla@yahoo.com.mx

**Keywords:** β-cells, *Carica papaya*, diabetes, pancreatic islets

## Abstract

The aim of the present study was to investigate the effect of *C. papaya* L. leaf extract (CPLE) on pancreatic islets in streptozotocin (STZ)-induced diabetic rats, as well as on cultured normal pancreatic cells with STZ in the medium. CPLE (3–125 mg/Kg) was administered orally for 20 days, while a group of diabetic rats received 5 IU/Kg/day of insulin. At the end of the treatment the rats were sacrificed. Blood was obtained to assess glucose and insulin levels. The pancreas was dissected to evaluate β cells by immunohistochemistry. In addition, normal pancreatic cells were cultured in a medium that included CPLE (3–12 mg). One half of the cultured cells received simultaneously CPLE and STZ (6 mg), while the other half received CPLE and five days later the STZ. After three days of incubation, insulin was assayed in the incubation medium. The CPLE administered to diabetic rats improved the fasting glycemia and preserved the number and structure of pancreatic islets. However, when CPLE was added to pancreatic cells in culture along with STZ, the insulin concentration was higher in comparison with the cells that only received STZ. In conclusion, the CPLE preserves the integrity of pancreatic islets, improves the basal insulin secretion and protects cultured cells from the adverse effects of STZ.

## 1. Introduction

Diabetes mellitus (DM) is a common endocrine disorder that involves the loss of pancreatic β-cell function; this fact is driving research into potential replacement sources [1]. Moreover, it is known that insulin is the only true specific marker identified for β-cells; the insulin levels reflect accurately the β-cell function but not necessarily the cell mass [1]. Therefore, an effective therapeutic strategy is desirable to prevent or delay the development of pancreatic β-cell dysfunction or death. More recently, studies in rodents have suggested that β-cells can be regenerated after being seriously damaged with streptozotocin treatment [2,3]. There are some studies underway trying to find natural substances that are effective in reducing the intensity of diabetes. In this sense, several natural compounds can suppress the activity of certain enzymes involved in glucose production and absorption, and some of them mimic the action of insulin [4]. Other studies have shown that certain compounds present in plants can modulate β-cell apoptosis and enhance the action of insulin [5].

*C. papaya* L. leaves have been used for treating nervous pain, gastric ulcers, malaria, asthma and as a vasodilator [6,7,8]. In addition, previous reports showed that in diabetic rats treated with *Carica papaya* leaf extract , the serum glucose levels decreased [9]. Therefore the objective of this study was to determine the effects of *C. papaya* extract on the number and integrity of β-cells in streptozotocin-induced diabetic rats, and also on the insulin secretion of normal pancreatic cells incubated with and without STZ in the culture medium.

## 2. Materials and Methods

### 2.1. Chemical Compounds and Plant Subsection

All chemicals were purchased from Sigma (St Louis, MO, USA). Other analytical grade reactants were obtained from Merck (Mexico City, Mexico). Kits for different enzyme assays were purchased from Biosystems S.A. (Barcelona, Spain). The *Carica papaya* leaves were collected from June to September 2012 from Cintalapa, in the state of Chiapas, Mexico. The plant was authenticated as *Carica papaya* at the Academic Division of Biological Sciences at the Juarez Autonomous University of Tabasco. A voucher specimen is kept in the herbarium (No. 32307) of this institution.

### 2.2. Preparation of Carica Papaya Leaf Extract

The leaves of *C. papaya* were washed with tap water and cut into small slices. The slices were powdered after air-drying. A 100 g dry sample was placed in a Soxhlet system and extracted for 8 h with 500 mL of chloroform. Subsequently, the solvent was evaporated under vacuum until the extract was completely dried, then it was stored at −20 °C. Ten g of semi-solid mass was obtained.

### 2.3. Animals

Experiments were performed in adult male Wistar rats (body weight range: 250–300 g), 10 to 11 weeks of age. Animals were housed and maintained at 22 °C under a 12-h light/12-h dark cycle, with free access to food and water. Experiments were carried out during the normal light/dark cycle and always started at the same time (10:00 AM). All experiments complied with the Guidelines on Ethical Standards for investigation in animals; the study was approved by the local Internal Committee for the care and use of laboratory animals (003-10/CICUAL/DACS) of the UJAT Academic Division of Health Sciences (initials in Spanish DACS).

### 2.4. Induction of Diabetes

Experimental diabetes [10] was induced following an overnight fast, by a single intraperitoneal injection of 60 mg/kg STZ freshly dissolved in distilled water. It is known that intraperitoneal injection of STZ leads to β-cell destruction in the pancreatic islets. This produces an insulin deficiency and increased blood glucose levels [11]. Control animals received 0.9% sterile saline. Hyperglycemia was confirmed four days after injection by measuring the tail vein blood glucose level with an Accu-Check Sensor Comfort glucometer (Roche, Mexico City, Mexico). Only the animals with fasting blood glucose levels ≥ 250 mg/dL were included in the study. Not a single rat was rejected in this study lot.

### 2.5. Experimental Design

A total of 56 rats (40 diabetic, 16 normal) were included in the study and were divided into seven groups of eight animals each:

Group 1:  Normal control (NC).
Group 2:  Normal rats that received 62 mg/kg/day of CPLE (NC + CPLE62)
Group 3:  Diabetic control (DC)
Group 4:  Diabetic rats that received 31 mg/kg/day of CPLE (D + CPLE31)
Group 5:  Diabetic rats that received 62 mg/kg/day of CPLE (D + CPLE62)
Group 6:  Diabetic rats that received 125 mg/kg/day of CPLE (D + CPLE125)
Group 7:  Diabetic rats that received 5 IU/kg/day of insulin (D + I)

The CPLE was given daily by oral gavage in 300 μL of polyethylene glycol (PEG). After 20 days of treatment, the animals were sacrificed by decapitation and blood was collected to perform biochemical serum analysis. The pancreas was obtained for histological and immunological examination.

### 2.6. Determination of Fasting Blood Glucose and Insulin

Blood was collected and the serum was immediately frozen and stored at −70 °C until analyzed for glucose and insulin. The serum glucose levels were analyzed using a Clinical Chemistry System ERBA XL200-Mannheim (Mannheim, Germany). Plasma insulin concentration was determined by an enzymatic immunoassay method (rat insulin ELISA, Bertin-Pharma, Montigny Le Bretonneux, France).

### 2.7. Isolation of Islets and Insulin Assay

Islets were obtained from the pancreas of a 200 g male Wistar rat. Immediately after isolation, the pancreas was cut in small fragments that were washed with Hank’s balanced salt solution (HBSS). The pancreatic cells were disaggregated by trypsinization; then the cells were collected by centrifugation and incubated during three days in culture chambers on Petri plates with enriched RPMI 1640 (Roswell Park Memorial Institute, Gibco; Grand Island, NY, USA) medium, 37 °C, 5% CO_2_. Cells (1 × 10^6^) were placed in 64-well plates with 100 mL of RMPI medium. The next day a volume of 75 µL was removed from each well and 100 µL of medium was added to maintain the cell growth; this partial change of medium was done for three consecutive days [12]. The cells were then incubated after the addition of CPLE (3, 6, 12 mg in 30 µL PEG) and glucose (2 g/L); one half of the wells received simultaneously the respective dose of CPLE and 6 mg of STZ in 30 µL of PEG. The other wells received the same dose of SZT five days after the administration of CPLE. A group of wells with pancreatic cells with no addition of neither CPLE nor SZT (positive control), and cells with only 6 mg of STZ added in 30 µL PEG (negative control) were included. After three more days of incubation, the cell growth was stopped by freezing the plate and the insulin released into the medium was quantified (Bertin Pharma rat insulin ELISA).

### 2.8. Histopathological Studies

The rat pancreas was isolated, washed and placed in 5% formaldehyde solution. After its inclusion in paraffin, sections of 5 µm thickness were mounted on slides and stained with hematoxylin and eosin (HE) for microscope observation. The histopathological analysis to measure the islet area (μm^2^) and diameters (μm) were calculated by the islet number and islet area per field [13].

### 2.9. Immunohistochemical Analysis

The pancreatic tissue was fixed in neutral buffered formaldehyde, then it was embedded in paraffin and sectioned at 5 μm thickness. The tissue slices were fixed on microscope slides and the paraffin was eliminated with a xylene rinse. The slices were dehydrated, washed twice with absolute alcohol and two more times with 95% ethanol. The tissue section was placed in 0.3% hydrogen peroxide in methanol with 5% plasma bovine serum for 20 min at room temperature to block the endogenous peroxidase and non-specific binding sites for antibodies. For the detection of insulin, the sections were incubated with a polyclonal guinea-pig anti-insulin (1:100 diluted PBS; phosphate buffered saline) antibody for 24 h, washed three times with PBS and then with a mouse anti guinea pig biotinylated antibody (1:500) for 30 h. After three washes the sections were incubated with the streptavidin-biotin-peroxidase complex. After 30 min of incubation, the sections were washed again with PBS and the excess of buffer was removed from the slides; subsequently were incubated with peroxidase/diaminobenzidine (DAB) for 3–5 min at room temperature and washed with distilled water. Finally, the slices were stained with hematoxylin, dehydrated and mounted in glycerin-gelatin [14]. The stained pancreas sections were used to measure the islet dimensions and the positive immunoreactive area (μm^2^) to insulin, as well as the integrated optical density (IOD) which is the summation of total pixel optical density, employing Image Pro Plus 7.0 program (Ei80 microscope, Nikon; Tokyo, Japan) [13]. 

### 2.10. Statistics Analysis

One-way analysis of variance (ANOVA) followed by Student-Newman-Keuls test were used to compare differences between treatments. Values were considered statistically significant when *p* < 0.05.

## 3. Results

### 3.1. Determination of Fasting Blood Glucose and Insulin

All SZT-treated rats exhibited hyperglycemia (344.5 ± 95.5 mg/dL) 4 days after the injection, in comparison with non-diabetic rats (70.4 ± 4.9 mg/dL). The oral administration of CPLE (31 and 62 mg/kg) significantly decreased fasting blood glucose levels (122.2 ± 36.1 mg/dL and 113.8 ± 25.1 mg/dL; respectively) in comparison with the diabetic control group (*p* < 0.05). The diabetic rats (DC group) showed a decrease of the serum insulin levels (0.58 ± 0.09 ng/mL) with respect to non-diabetic rats (1.1 ± 0.12 ng/mL). However, the treatment with CPLE did not modify the insulin concentration in STZ-treated rats. On the other hand, a significant increase of serum insulin levels was observed in non-diabetic rats that received CPLE 62 mg/kg (1.9 ± 0.26 ng/mL) compared to the NC group.

### 3.2. Insulin Basal Release by the Pancreatic Islets

We observed that cultured pancreatic cells treated with STZ (negative control) reduced significantly their insulin basal release (0.93 ± 0.44 ng/mL) into the incubation medium which contained glucose (2 g/L), compared to cells with no additions (positive control; 9.76 ± 1.07 ng/mL) (Table 1). When CPLE (6 mg) was added to cells with no STZ, they showed a higher insulin release (11.9 ± 1.07 ng/mL) in comparison with both positive and negative controls (*p* < 0.05). Furthermore, when STZ was added simultaneously with the CPLE (3, 6 or 12 mg) the insulin release decreased in all three conditions but not in a dose-dependent manner (Table 1). However, when STZ was added five days after the CPLE, the insulin released by the islets was higher than that from normal control cells (positive control; 9.76 ± 1.07 ng/mL) (*p* < 0.05). and similar to that from normal cells with 6 mg of CPLE (Table 1).

### 3.3. Islets Microscopy and Insulin Immunohistochemistry

**Figure 1:**
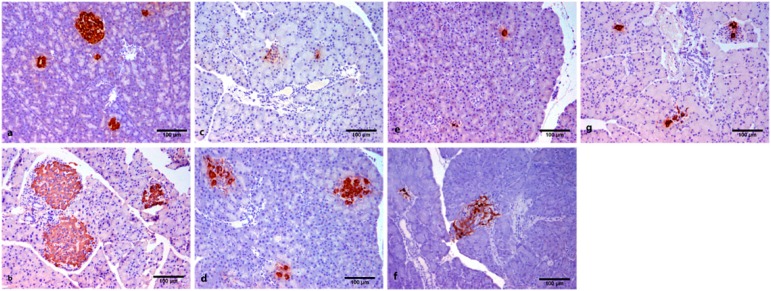
Pancreas histopathological examination and insulin immunohistochemistry from diabetic rats treated with *C. papaya* extract. (**a**) Normal control (NC) β-cells from normal islet with a greater content of insulin; (**b**) Normal rats that received 62 mg/Kg/day of CPLE (NC + CPLE62), large islets have greater insulin content when compared to NC; (**c**) Diabetic (D), β-cells from small islets have a significantly reduced insulin-positive area; (**d**) Diabetic rats that received 31 mg/Kg/day of CPLE (D + CPLE31), small islets have a greater density of insulin granules than β-cells from D; (**e**) Diabetic rats that received 62 mg/Kg/day of CPLE (D + CPLE62), showed a significant increase in positive insulin immunoreactive with respect to pancreas from D; (**f**) Diabetic rats that received 125 mg/Kg/day of CPLE (D + CPLE125), diffuse islet which has a low density of insulin granules; (**g**) Diabetic rats that received 5 IU/kg/day of insulin display islets with bigger insulin content.

The pancreatic islet area and diameter in diabetic rats were bigger in the CPLE-treated groups when compared with diabetic rats (*p* < 0.05, Table 2). The islet area and diameter in non-diabetic rats decreased compared with N + CPLE62 (*p* < 0.05, Table 2, Figure 1a,b). We showed that diabetic pancreas had a significantly reduced insulin-positive area (225.4 ± 107.2 µm^2^) with respect to normal control (1690 ± 286.4 µm^2^ , Table 2). In addition, diabetic rats treated with CPLE (31 mg/kg) showed a significant increase in positive insulin immunoreactive area (420.5 ± 144.4 μm^2^) when compared with diabetic control (225.4 ± 107.2 μm^2^, Table 2). The pancreas from non-diabetic rats with CPE62 showed an insulin immunoreactive area (1077 ± 762.2 µm^2^) similar to normal control rats (1690 ± 286.4 µm^2^). In general, the insulin positive area and the integrated optical density (IOD) was higher in CPLE-treated rats (31 mg/kg, 420.5 ± 144.4 μm^2^ and 298 ± 125.1, respectively, Figure 1d) when compared to the pancreas from diabetic rats (225.4 ± 107.2 μm^2^ and 164.9 ± 94, Figure 1c), but the insulin positive area and IOD was similar in CPLE-treated rats (62 mg/kg) with respect to diabetic control (Table 2, Figures 1f,c, respectively). However, diabetic rats treated with CPLE (125 mg/kg) showed a significant decreased a labeled area (59.8 ± 9.3 µm^2^) and IOD (29.3 ± 5.5) when compared with diabetic rats (Figures 1f,c, respectively, Table 2). In addition, diabetic rats treated with insulin showed an immunoreactive area (373 ± 169 µm^2^) and IOD (289 ± 93) similar to diabetic rats treated with *C. papaya* (31 mg/kg) (Table 2) (Figure 1g,d; respectively).

## 4. Discussion

### 4.1. Determination of Fasting Blood Glucose and Serum Insulin

The use of CPLE decreased the high fasting glucose levels in diabetic rats after the three different doses (31, 62 and 125 mg/kg). The administration of chloroform extract of *C. papaya* leaf did not increase the concentration of serum insulin in diabetic rats. These data suggest that the hypoglycemic effect of *C. papaya* is not mediated by the insulin pathway, since we used a model of diabetes with STZ that develops defective insulin secretion (because the pancreatic islets are destroyed by STZ injection) [11]. Furthermore, no decrease of glucose levels was observed when 125 mg/kg of chloroform extract of *C. papaya* was added; we also observed that this extract had a high viscosity and at this concentration it was not dissolved, which prevented the intestinal absorption of secondary metabolites. There is evidence that some plant phytoconstituents decrease fasting glycemia [15,16]. Furthermore, some plant secondary metabolites stimulate enzymes involved in the carbohydrate metabolism (disaccharides), increasing glycogen storage in the liver, glucose uptake by the muscle, inhibit gluconeogenesis, favor triglyceride synthesis and storage in the adipose tissue, and inhibit protein synthesis; altogether these effects correct the altered carbohydrates and fatty acids metabolism associated with diabetes, leading to a postprandial glucose control [15,17].

### 4.2. Insulin Basal Release by the Pancreatic Islets

The results with the use of CPLE suggest a protective action on pancreatic islets that agree with other studies employing natural products which can promote the regeneration of pancreatic β-cells and improve insulin function [17]. Other studies propose that medicinal plants possibly act stimulating the insulin secretion from pancreatic β-cells *in vivo* and *in vitro* [16,18]. However, in the present study the CPLE treatment did not modify the blood insulin levels of animals.

### 4.3. Effect of C. papaya on Histopathological and Insulin Immunohistochemistry Studies

The islet area and diameter were bigger in CPLE-treated groups when compared with the islets from diabetic control rats. In addition, the treatment with CPLE decreased hyperglycemia, increased insulin secretion in cultured pancreatic cells and preserved pancreatic β cells. These effects may be a consequence of the protection of β-cells from oxidative stress. Some plant sterols decrease the blood glucose through an insulin secretion improvement, potentiation, proliferation or accumulation of β-cells [19,20]. Besides, some other studies suggest that plant secondary metabolites promote regeneration of β-cells in the pancreas [21,22], and certain steroids stimulate the islets to secrete and release insulin *in vitro* [23,24].

Other reports indicate that extracts from natural products can protect β-cells by enhancing the islets function and restoring β cell mass [25]. In addition, the proliferation and preservation of β-cell mass and function will be the better way to prevent diabetes [19,20,22]. However, in this study we showed that insulin positive area and the integrated optical density (IOD) was higher in CPLE-treated rats (31 mg/kg), but not in CPLE-treated rats (61 and 125 mg/kg). In this sense, recently it has been suggested that this regeneration might be through transdifferentation of β-cells that undergo a conversion into other endocrine cell types, providing a reciprocal relationship between β-cells and α-cells in diabetes [3]. The CPLE shows a protective effect on the β-cell damage induced by STZ, but the extract does not have a regenerating action on pancreatic cells as we observed no increase in serum insulin.

On the other hand, a previous study showed that aqueous extract of *C. papaya* prevents oxidative stress in diabetic rats through the increased of serum nitric oxide levels [26]. As it is well known, the physiological role of an antioxidant is to prevent damage to cellular components arising as a consecuence of chemical reactions involving free radicals [26,27]. Besides, diabetes is characterized by hyperglycemia and hyperlipidemia, two biochemical features associated with inhibition of endothelial nitric oxide synthase (eNOS), leading to diminished NO production, increased formation of reactive oxygen species (ROS) and formation of free radicals, lower efficacy of antioxidant systems , that are important agents in the development of diabetic complication [27,28]. In this study we show that the administration of *C. papaya* under diabetic conditions induced an improvement in hyperglycemia and in β-cell function, we suggested that these effects are associated with the antioxidative propeties of the extract. In that sense, interestingly a number of chemicals of plant extracts possess potent activities, both *in vitro* and *in vivo* to prevent oxidative stress restoring mass of β cells and its structure by reducing damage of pancreatic islets in diabetic rats [18,28].

Our results are in line with those reported by Gogna *et al.* [29] that show an antioxidant capacity of *C. papaya* leaves on C2C12 cell line and free radical scavenging activity. The nuclear magnetic resonance spectroscopy analysis of *C. papaya* leaf showed the presence of a large number of metabolites (phenylpropanoid esters, flavonoids, quinate, quinic acid, coumarate, caffeate, naringenin and quercetin) known for their antioxidant activity [29]. The plant can exert its action by increasing the proliferation or the renewal of the islet β-cells following destruction by STZ. This is clearly reinforced by the strong immunostaining of islet β-cells of rats treated with the plant extract. In this sense, there is evidence that the pharmacological effects of phytomedicine can be attributed to the synergistic action of several plant constituents [18,19,28,29].

One of the limitations of this study is that we did not employ any pancreatic regenerative cell markers. It remains to be determined which mechanisms are accountable for the present observations and should be considered for future studies.

## 5. Conclusions

The CPLE reduced the fasting glycemia of diabetic rats. The extract also preserved the number, morphology and immune response to insulin antibodies of pancreatic islets. The addition of CPLE protected the pancreatic cells from the adverse action of SZT, especially when this drug was added several days after the cells were incubated with CPLE. Further studies are needed to understand the mechanism behind these findings.

## Figures and Tables

**Table 1:** Effect of the chloroform extract of *C. papaya* on insulin release from cultured β-cells.

Group	Insulin (ng/mL)	Insulin (ng/mL) STZ (6 mg) Added Simultaneously with CPLE	Insulin (ng/mL) SZT (6 mg) Added 5 Days after CPLE
None	9.76 ± 1.07		
SZT	0.93 ± 0.44 ^a^		
CPLE	11.9 ± 1.07 ^b^		
CPLE 3 mg + SZT		5.26 ± 0.30 ^a^	12.47 ± 0.16 ^a,c^
CPLE 6 mg + SZT		2.70 ± 0.69 ^a^	12.70 ± 0.15 ^a,c^
CPLE 12 mg + SZT		0.95 ± 0.20	12.85 + 0.20 ^a,c^

Data are expressed as mean ± S.E.M.; *n* = 6. One-way analysis of variance (ANOVA) followed by Student-Newman-Keuls test (*p* < 0.05). None (positive control); β-cells culture without STZ, *Carica papaya* leaf extract (CPLE). STZ (negative control); cells with STZ. ^a^ Statistically different from β-cells culture without streptozotocin (None; positive control); ^b^ Statistically different from β-cells culture with streptozotocin (STZ; negative control) (*p* < 0.05)*.* ^c^ Statistically different from β-cells culture with STZ and CPLE added simultaneously (*p* < 0.05).

**Table 2:** Effect of the chloroform extract of *C. papaya* on the Pancreas islet.

Group	Islet Area (μm^2^)	Diameter (μm)	Labeled Area (μm^2^)	IOD
N	2283 ± 263.2	93.9 ± 4.1	1690 ± 286.4	1386.8 ± 231.1
N + CPLE62	11,410 ± 4116 ^a^	246.5 ± 56.2 ^a^	1077 ± 762.2	688 ± 554.8 ^a^
D	457.8 ± 116.9 ^a^	41.3 ± 18.5 ^a^	225.4 ± 107.2 ^a^	164.9 ± 94 ^a^
D + I	17,044 ± 2420 ^b^	308.4 ± 59.6 ^b^	373 ± 169 ^b^	289 ± 93 ^b^
D + CPLE31	17,300 ± 5390 ^b^	330.7 ± 77.5 ^b^	420.5 ± 144.4 ^b^	298 ± 125.1 ^b^
D + CPLE62	17,787 ± 4782 ^b^	335 ± 66.7 ^b^	269.4 ± 62.5	165.3 ± 34.0
D + CPLE125	8850 ± 1183 ^b^	212.2 ± 11.5 ^b^	59.8 ± 9.3	29.3 ± 5.5

Analysis by Image Pro Plus 7.0 Program and data are expressed as mean ± S.E.M. One-way analysis of variance (ANOVA) followed by Student-Newman-Keuls test (*p* < 0.05). IOD (integrated optical density), N (normal control; NC), D (diabetic control; DC). ^a^ Statistically different from normal control (NC); ^b^ Statistically different from diabetic control (DC). (*p* < 0.05)*.*

## Abbreviations

The following abbreviations are used in this manuscript:

DM	Diabetes mellitus
CPLE	*Carica papaya* leaf extract
NC	Normal control
D	Diabetic
STZ	Streptozotocin
IOD	Integrated optical density
HE	Hematoxylin and eosin
PEG	Polyethylene glycol
DAB	Diaminobenzidine
PBS	Phosphate buffered saline
HBSS	Hank’s balanced salt solution
